# Rabies Encephalitis and the Use of Optic Nerve Sheath Diameter to Detect Elevated Intracranial Pressure

**DOI:** 10.7759/cureus.32154

**Published:** 2022-12-03

**Authors:** Dan Arreaza Kaufman, Eva Teng, Nicolas Biro

**Affiliations:** 1 Ophthalmology, New York Medical College - Jamaica Hospital Medical Center, New York, USA

**Keywords:** intracranial pressure, optic nerve, optic nerve sheath diameter, encephalitis, rabies

## Abstract

Rabies is a rare but rapidly progressive and almost universally fatal disease. A previously healthy 59-year-old male presented with rabies encephalitis. We measured his optic nerve sheath diameter (ONSD) daily in both eyes using ultrasonography to indirectly monitor for elevated intracranial pressure (ICP). We performed CT and MRI brain on days when his ONSD changed significantly. An increase in ONSD temporally correlated with radiologic findings of cerebral edema and acute subarachnoid hemorrhage (SAH). ONSD measurement is a fast, inexpensive, and widely-available imaging modality that may serve as a surrogate marker for elevated ICP. It may be especially useful in patients who are difficult to be transported to radiology due to the unstable nature of their disease.

## Introduction

Rabies is a viral disease transmitted by the bite of an infected animal, and it causes encephalitis in humans and other mammals. Without post-exposure prophylaxis, most human patients develop symptoms of rabies 20-90 days after exposure, and these include flu-like symptoms, headaches, confusion, paresthesias, excess salivation, and agitation [1]. Rabies encephalitis may present with significant inflammation and cerebral edema [2]. Obtaining neuroimaging studies can be challenging and time-consuming since patients often deteriorate rapidly and can become agitated.

## Case presentation

A previously healthy 59-year-old male presented to the emergency department with a two-day history of nausea, vomiting, and agitation. He had traveled to the Philippines six months prior where he had been bitten by a stray dog, and he had not received the rabies vaccine or post-exposure prophylaxis. Within 12 hours of admission, he became tremulous and combative and developed hypersalivation. Immunofluorescent antibody testing of skin biopsy and saliva confirmed rabies virus infection. The baseline eye exam showed normal anterior and posterior segments, with a cup-to-disc ratio of 0.3 and no sign of disc edema in both eyes. He was intubated on day four and placed on the Milwaukee protocol, which consisted of sedation using midazolam, haloperidol, and ketamine to reduce neuronal hyperexcitation while awaiting viral clearance by the immune system [3].

**Figure 1:**
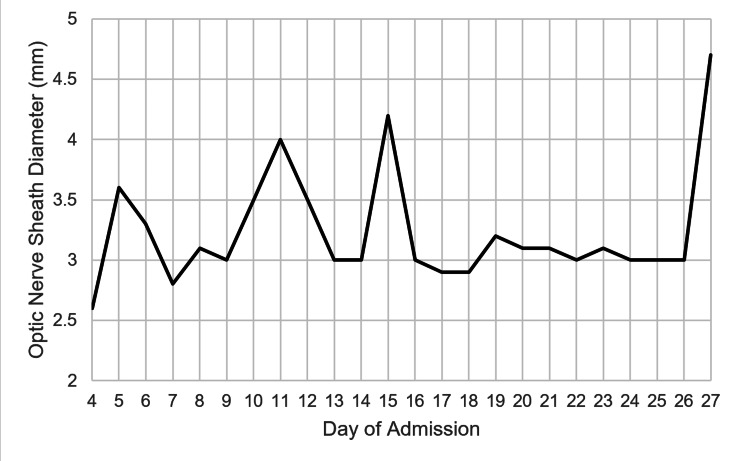
Averaged optic nerve sheath diameter (mm) during the hospital course

Starting on day four, his optic nerve sheath diameter (ONSD) was measured daily in both eyes using ultrasonography (Sonosite II Ultrasound System, FUJIFILM SonoSite, Bothell, WA) to indirectly monitor for elevated intracranial pressure (ICP). The diameters of the right and left eyes were averaged and the trend was examined daily (Figure 1).

CT head and MRI brain were performed on days with significant changes in ONSD.

On day nine of admission, the patient developed anisocoria, while his ONSD and CT head showed no acute changes. On day 11, his ONSD increased to 4.0 mm, and the CT head revealed acute infarction of the right globus pallidus and internal capsule with subarachnoid hemorrhage (SAH) anterior to the right frontal lobe. MRI brain showed petechial hemorrhage with surrounding cytotoxic edema in the bilateral medulla and in the right internal capsule. MRI angiogram identified narrowing along M1 and P1 segments and basilar artery, concerning for infection-related vasospasms. The ONSD then decreased to 3.0 mm on day 13, and the corresponding CT head showed a decreased size of the SAH. Between days 14 and 15, the patient's ONSD increased from 3.0 to 4.2 mm, a 40% rise, while his pupils became fixed and dilated. Repeat brain imaging revealed stable SAH. The patient was weaned off sedation with no improvement in his neurological status. On day 27, his ONSD increased from 3.0 to 4.7 mm, a 55% rise from the previous day. MRI brain showed new edema involving the gray matter structures. Given these findings, the patient's poor neurological function, and grave prognosis, the family decided to withdraw supportive care and the patient died shortly after.

## Discussion

The rabies virus belongs to the Lyssavirus genus in the Rhabdoviridae family. It is usually transmitted by the bite of an infected animal, and it enters the peripheral nerves and is then transported to the central nervous system by retrograde axoplasmic transport [4]. The stages of rabies include incubation, prodrome, acute neurological signs, coma, and death. Neuroimaging in the prodromal and acute neurological phase with MRI may show abnormal hypersignal T2 changes in the spinal cord, temporal lobe cortices, hippocampal gyri, and cerebral white matter [1]. However, it may be difficult to obtain neuroimaging studies in the acute neurological phase as patients may be agitated, uncooperative, or have fluctuating consciousness.

The optic nerve sheath (ONS) is anatomically continuous with the dura mater, and increased ICP leads to distended ONS. ONSD measurement using ultrasonography is a fast, inexpensive, non-invasive, and widely available imaging modality that may serve as a surrogate marker for elevated ICP. It has been used to detect increased ICP in meningitis, traumatic brain injury, and ischemic stroke [5,6]. Daily ONSD measurement in this patient with rabies encephalitis correlated temporally with CT and MRI findings of cerebral edema and acute SAH. This case illustrates the potential for using ultrasound to non-invasively measure ICP in the ICU setting, especially in patients who are difficult to be transported to radiology due to the unstable nature of their disease. It may also be used to indirectly monitor ICP when direct ICP measurement techniques such as CSF ventricular catheter are unavailable or contraindicated.

Potential limitations of this method include inter- and intra-operator variability, quality of ultrasound machine used, and inexperience with ocular sonography [7]. In our case, the data were collected mainly by one operator. ONSD measurement also provides an indirect approximation of ICP, and it cannot be converted to exact ICP values. While impractical for this particular case, future studies may try to correlate ONSD with direct ICP measurement. This could potentially be done in patients with idiopathic intracranial hypertension as they often undergo ICP measurement as part of their workup.

## Conclusions

Rabies encephalitis is a rapidly progressive disease, and performing neuroimaging studies can be challenging in this patient population. While the prognosis is invariably poor, patients are commonly placed on the Milwaukee protocol and closely monitored in the ICU for neurological complications. ICP may be elevated in rabies, and ONSD measurement using ultrasonography may serve as a surrogate marker for elevated ICP. Clinicians may consider using ONSD and its trend as a point-of-care guide on when to obtain further neuroimaging. Further studies are required to better correlate ONSD and ICP values.

## References

[1] Hemachudha T, Ugolini G, Wacharapluesadee S, Sungkarat W, Shuangshoti S, Laothamatas J (2013). Human rabies: neuropathogenesis, diagnosis, and management. Lancet Neurol.

[2] Fooks AR, Banyard AC, Horton DL, Johnson N, McElhinney LM, Jackson AC (2014). Current status of rabies and prospects for elimination. Lancet.

[3] McDermid RC, Saxinger L, Lee B, Johnstone J, Gibney RT, Johnson M, Bagshaw SM (2008). Human rabies encephalitis following bat exposure: failure of therapeutic coma. CMAJ.

[4] Tsiang H (1979). Evidence for an intraaxonal transport of fixed and street rabies virus. J Neuropathol Exp Neurol.

[5] Stead GA, Cresswell FV, Jjunju S, Oanh PK, Thwaites GE, Donovan J (2021). The role of optic nerve sheath diameter ultrasound in brain infection. eNeurologicalSci.

[6] Güzeldağ S, Yılmaz G, Tuna M, Altuntaş M, Özdemir M (2021). Measuring the optic nerve sheath diameter with ultrasound in acute middle cerebral artery stroke patients. J Stroke Cerebrovasc Dis.

[7] Geeraerts T, Launey Y, Martin L, Pottecher J, Vigué B, Duranteau J, Benhamou D (2007). Ultrasonography of the optic nerve sheath may be useful for detecting raised intracranial pressure after severe brain injury. Intensive Care Med.

